# Current Opinions in Open and Endovascular Treatment of Major Arterial Injuries in Pediatric Patient

**DOI:** 10.3390/jcm12154906

**Published:** 2023-07-26

**Authors:** Marco Franchin, Paolo Righini, Mario D’Oria, Daniela Mazzaccaro, Giovanni Nano, Matteo Tozzi, Gabriele Selmo, Gabriele Piffaretti

**Affiliations:** 1 Vascular Surgery, Department of Cardio-Thoracic and Vascular Surgery, ASST Settelaghi University Teaching Hospital, 21100 Varese, Italy; marco.franchin@hotmail.it; 2 Vascular Surgery–IRCCS Policlinico San Donato, 20097 San Donato Milanese, Italy; paolo.righini@grupposandonato.it (P.R.); daniela.mazzaccaro@grupposandonato.it (D.M.); giovanni.nano@unimi.it (G.N.); 3 Vascular Surgery, Cardiovascular Department, University Hospital of Trieste ASUGI, 34129 Trieste, Italy; mario.doria88@outlook.com; 4 Vascular Surgery, Department of Medicine and Surgery, University of Insubria School of Medicine, 21100 Varese, Italy; matteo.tozzi@uninsubria.it; 5 Anesthesia and Palliative Care, Department of Anesthesia and Intensive Care, ASST Settelaghi University Teaching Hospital, 21100 Varese, Italy; gabriele.selmo@asst-settelaghi.it

**Keywords:** pediatric arterial injury, pediatric vascular trauma, upper extremity arterial injury, lower extremity vascular injury

## Abstract

Pediatric major arterial vascular injuries may belong to the same principal categories as adults, but have been poorly documented, with an estimated overall incidence of <2% of all vascular traumas. Open surgery has been the mainstay of treatment, but no clear guidelines have been developed to recommend the best practice patterns in terms of strategy or repair as well as postoperative pharmacological regimen. Herein, we report three cases and a narrative review of the available literature regarding the main aspects when dealing with pediatric arterial injuries based on the predominant series available from the most recent published literature.

## 1. Introduction

Pediatric major arterial vascular injuries may belong to the same principal categories as adults, but over the course of the last decades, they have been poorly documented [1]. First, there is not a commonly accepted threshold to define a pediatric vascular injury, but according to most of the published literature, it should be defined as a vascular injury occurring in patients between 2 and 16 years of age [2]. Second, the true incidence is still matter of debate, since most reported experiences primarily concerned upper and lower extremity injuries, while lesions of the supra-aortic trunks or of the torso (e.g., thorax and abdomen) were rarely included [3,4,5,6,7,8,9,10]. Last, open surgery has been the mainstay of treatment, but no clear guidelines have been developed to recommend the best practice patterns in terms of strategy or repair as well as postoperative pharmacological regimen. Herein, we report three cases from our experience, as well as a summary of the available literature regarding the main aspects of dealing with pediatric arterial injuries based on the predominant series available from the published literature.

## 2. Methods

For this review, the SANRA (Scale for the Assessment of Narrative Review Articles), a six-item scale developed for the quality assessment of narrative review articles, was adopted as an optimal presentation of selected studies [11]. Although SANRA is usually used during the peer-review process, the authors tried to obtain the maximum score possible (12 points) to improve the quality of our review. Furthermore, recommendations from Green and collaborators were adopted [12]. The results are presented in a narrative form.

## 3. Search Strategy

The research was conducted on PubMed (MEDLINE); only English-language articles were evaluated with at least 10 cases per series included. Keywords were selected using medical subject headings for PubMed; keywords, such as “pediatric vascular trauma”, “non-iatrogenic vascular trauma”, “vascular injury”, and “arterial vascular extremity trauma” were used and combined to obtain the first publication cluster. The Boolean operators “AND” and “OR” were used for connecting terms with each other. All titles and abstracts of potentially useful articles were selected. Two researchers (G.P., M.F.) independently screened titles, abstracts, and full texts. In case of discrepancies in article or data extraction, a third researcher (P.R.) was consulted to provide the final judgment. Data from all included studies were then independently extracted. References of all identified relevant studies were used to perform a recursive search of the literature.

### 3.1. Institutional Case Report #1—Upper Extremity Injury and Open Surgery

**Figure 1:**
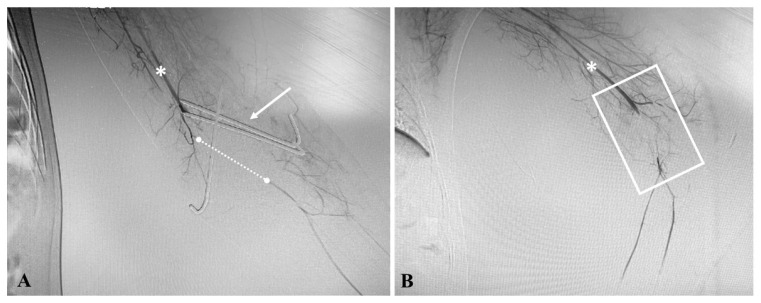
Patient #1: diagnostic angiography performed at non-academic non-teaching hospital after humer fracture fixation ((**A**), white arrow) showing the discontinuity ((**A**), dashed line; (**B**), white square) of the distal segment of the left brachial artery ((**A**,**B**), asterisk).

This 6-year-old female was referred to the Emergency Department after a bicycle fall at home. She was referred to another hospital where orthopedic surgeons fixed a homer fracture using a posterior approach. A few hours after this operation, due to the persistent pallor and hypothermia of the forearm, vascular evaluation was advised, and an angiography was performed from the right groin which documented the interruption at the most distal aspect of the brachial artery with the revascularization of both the radial and ulnar artery (Figure 1A,B).

**Figure 2:**
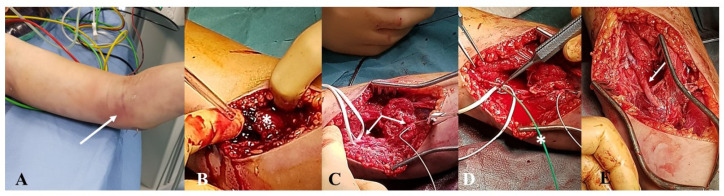
Patient #1: preoperative clinical evaluation of the antecubital expanding hematoma ((**A**), white arrow). Surgical exploration (**B**) revealed an extensive fresh hematoma (asterisk) determined by the transection of the distal brachial artery ((**C**), white arrows) which was clamped with endoluminal Fogarty catheter ((**D**), asterisk). Complete reconstruction with an autologous basilic vein ((**E**), white arrow).

No signs of anemia were observed and no neurological deficits were detected. Vasospasm was suspected and the clinical scenario managed with a conservative approach. The worsening of the clinical scenario was then characterized by the onset of a non-pulsatile mass at the antecubital fossa associated with the development of hand paralysis and forearm paresthesia (Figure 2A). The patient was transferred to our tertiary academic teaching hospital to prompt intervention; thus, in light of the previous angiography, a transection of the brachial artery was suspected and an immediate repair in the operating room was planned. At the surgical exploration, a complete transection of the distal aspect of the brachial artery was discovered which was repaired with an interposition graft using a short segment of the autologous basilic vein and interrupted suture using 7/0 polipropilene (Figure 2B–E).

The patient was discharged home on postoperative day 12 with major neurologic sequalae due to the delay in the diagnosis at the time of the angiography. At 36 months follow-up, the interposition graft was still patent with no anastomotic enlargement and the neurologic sequelae completely resolved after an intense rehabilitation program.

### 3.2. Institutional Case Report #2—Intra-Abdominal Injury and Endovascular Treatment

This 11-year-old male was referred to the Emergency Department after a high-speed trauma (motorbike driver against fixed obstacle). At the first medical examination at the scene, he was conscious (Glasgow Coma Score = 15) and hemodynamically unstable (blood pressure = 90/50 mmHg, heart rate = 120 ppm, Shock Index = 1.33). Blood tests showed hemoglobin was 9.8g/dL, base excess −2, lactate 18 mg/dL. While chest and pelvic X-rays did not show abnormalities, the computed tomography-angiography revealed the rupture of the right common iliac artery. Under general anesthesia and surgical exposure of the right common femoral artery, the arterial injury was successfully sealed off using a self-expandable covered stent (Viabahn^®^ 5 mm × 50 mm—W.L. Gore & ass; Flagstaff, AZ, USA). Intervention time was 28 min and blood loss was absent. Postoperative course was uneventful and he was discharged home on postoperative day 3 on single antiplatelet therapy. At 24 months follow-up, the patient was still alive and well with no disability; the duplex-ultrasound confirmed the patency of the covered stent.

### 3.3. Institutional Case Report #3—Aortic Injury and Open Surgery for Failed Endovascular Treatment

**Figure 3:**
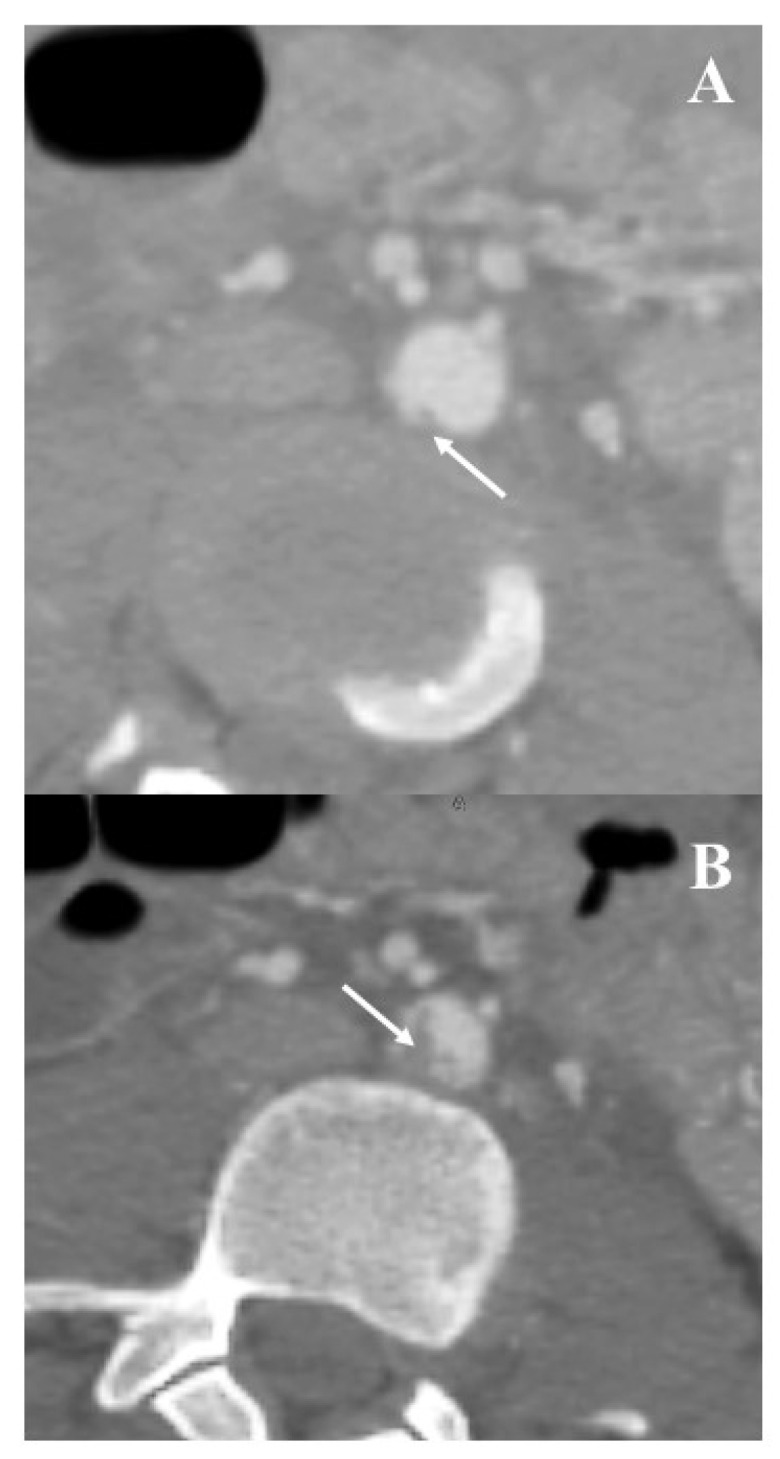
Patient #3: preoperative computed tomography-angiography showing the disruption of the intimal contour ((**A**), white arrow) with a large intimal flap ((**B**), white arrow).

This 15-year-old female patient was referred to the Emergency Department after motorway low-speed trauma (motor vehicle driver against fixed obstacle). At the first medical examination, she was conscious (Glasgow Coma Score = 15) and hemodynamically stable (blood pressure = 120/70 mmHg, heart rate 130 ppm, Shock Index = 1.08); no immediate signs of life-threatening lesions were detected. At arrival, a complete panel of blood tests was performed which did not reveal abnormalities, but the computed tomography-angiography showed a 10 mm abdominal aortic tear with a focal surrounding intramural hematoma (Figure 3A,B); it was a type 2 according to the Society for Vascular Surgery classification [11].

**Figure 4:**
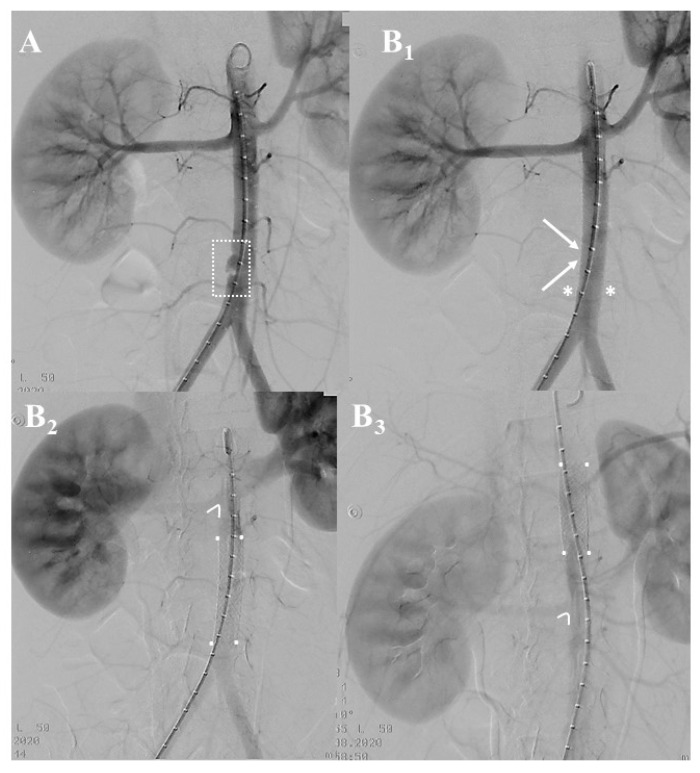
Patient #3: intraoperative angiography showing the aortic lesion ((**A**), white dashed square). The covered stent ((**B_1_**), white arrows) successfully sealed the aortic lesion as confirmed by the disappearance of the most distal lumbar arteries ((**B**), white asterisks). At the first confirmatory angiogram (**B_2_**) the covered stent (white dots) was correctly positioned in the infrarenal aorta (angled line). Completion angiogram (**B_3_**) unexpectedly documented the cranial displacement of the covered stent (white dots) that now was in the suprarenal aorta (angled line) at the level of the superior mesenteric artery.

Under general anesthesia, we sealed the lesion using an endovascular repair using a balloon-expandable covered stent (Vbx^®^ 11 mm × 59 mm—W.L. Gore & ass.; Flagstaff, AZ, USA). The deployment was uneventful (Figure 4(A,B_1_)). However, after the molding of the covered stent to optimize stent adaptation to the aortic size, the completion angiography unexpectedly showed the proximal migration of the covered stent at the level of the superior mesenteric artery (Figure 4(B_2_,B_3_)).

**Figure 5:**
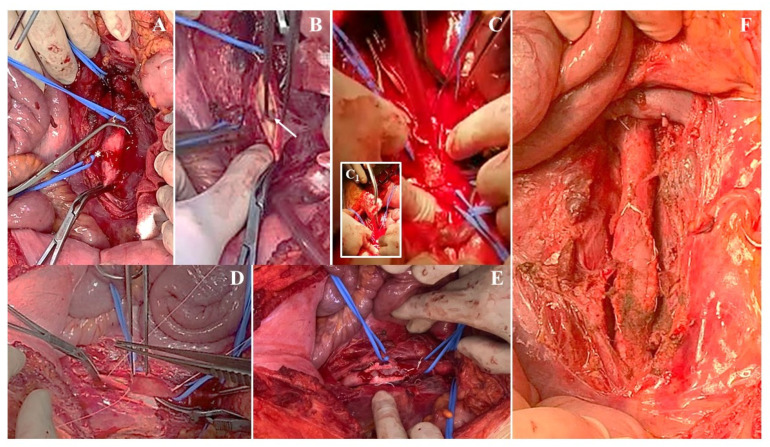
Patient #3: intraoperative findings. Surgical exposure of the infrarenal aorta (**A**) at the level of the inferior mesenteric artery (white asterisks). After longitudinal aortotomy (**B**) an ovale, postero-lateral lesion was confirmed (white arrow). The covered stent was explanted (**C**,**C_1_**) and the lesion repaired with a paricardial patch angioplasty (**D**) with posterior reinforcement (**E**) by means of a Teflon strip felt with a good final result (**F**).

An open conversion was performed to explant the covered stent after it was gently mobilized infrarenal with the use of an angioplasty balloon. The direct exploration of the aorta confirmed the focal defect with no interruption of the aortic wall and the lesion was repaired using a xeno-pericardium patch angioplasty posteriorly reinforced with a Teflon strip felt (Figure 5A–F).

No postoperative complications were observed and the patient was discharged home on postoperative day five on single antiplatelet therapy. At 36 months follow-up, the patient was alive and well with no disability; duplex-ultrasound confirmed the patency of the terminal aorta without prosthetic complication at the site of the aortic repair.

## 4. Results

### 4.1. The Amount of Problem

Pediatric vascular injuries are rare situations; the low incidence and specific anatomic and physiological characteristics may pose significant therapeutic challenge in terms of diagnostics, operative treatment, and perioperative management. There is a scarcity of reported experience for the management of such injuries; the true incidence of any vascular arterial injury among patients younger than 16 years is unknown. The results of our review on the largest experiences reporting on major arterial injuries in pediatric patients demonstrate a wide incidence ranging from 0.4–4.4%, depending on the type of injury/injuries analyzed (Table 1). Nevertheless, it has been homogeneously reported to be lower than in adults, overall [1,4]. These are the dominant reasons why guidelines with management strategies are lacking.

### 4.2. Presenting Patterns and Type of Injuries

Similar to what has been observed in adult patients, vascular lesions are grouped into three major classifications: iatrogenic, which occur as part of a diagnostic or therapeutic procedure; those that originate from a blunt trauma; and those that result from a penetrating trauma that involves partial or complete transection of the blood vessel [22]. The different robust experiences available in the literature are heterogeneous in reporting different trauma cohorts with a broad spectrum of different injury mechanisms, anatomic regions, and morphologic types of lesions. Nevertheless, our review found they were homogeneous in reporting a higher likelihood of penetrating trauma as a cause of vascular injury in the pediatric population as compared with blunt trauma. Barmpras et al. [1] analyzed the largest cohort so far, deriving from the National Trauma Databank, and observed that pediatric patients who sustained a vascular injury were, overall, also significantly less severely injured when compared to their adult counterparts. This finding has been explained by the predominant incidence of upper extremity vascular injuries among pediatric patients, and also was likely responsible for the lower overall crude mortality observed in the pediatric population when compared to adults. Upper extremity vascular injuries involving brachial, ulnar, and radial vessel injuries were most commonly encountered, with falls and stab wound injuries being the most frequent injury mechanisms. In contrast, despite the scant of data on aortic trauma, the Kids’ Inpatient Database, which comprises 468 injuries on a national level, showed that the most common mechanism of injury was motor vehicle-related [13].

### 4.3. Vascular Diagnostic

One of the most debated aspects of pediatric vascular trauma management is vascular diagnostic. The algorithm for pediatric vascular trauma management comes predominantly from the adult trauma literature. Previously, angiography was the “gold standard” for diagnosis of possible vascular injuries, especially in peripheral arterial lesions, but recently it has been supplanted by computed tomography-angiography in most experiences as a noninvasive and readily available diagnostic tool in pediatric patients as well. As solicited by Sciarretta et al. [4], all of those whom presented with obvious hard signs of vascular injury required immediate operation, and therefore, further diagnostic studies should be limited. This approach finds support in the experience of Mommsen et al. [3], whose majority of patients received no specific vascular diagnostics; this occurred especially in patients with supracondylar fractures, penetrating injuries, and isolated blunt extremity trauma. In contrast, vascular diagnostics were performed in >70% of patients with multiple traumas in order to avoid long surgical procedures with intraoperative evaluation of vascular injuries. 

### 4.4. Key Role of Pediatric Expertise

Owing to the low incidence rate, a robust clinical exposure to pediatric vascular injuries is unlikely for most surgeons [1,14,15,23]. Nevertheless, developing a skill set that encompasses pediatric physiology as well as open vascular surgical exposures and techniques during traumatic scenarios would be of extreme importance [1,16]. Considering that the operation of pediatric patients poses a significant challenge in terms of technical aspects and a significant amount of stress, maintaining humility and leveraging institutional collaborative expertise, as well as learning from those who care for children with regularity could be crucial [7,17].

### 4.5. Essentials of Open Operative Repair

There are no specific guidelines, protocols, or definitive recommendations regarding the optimal surgical approach in pediatric patients. Thus, the more reasonable determinant factors to lead management strategy have been identified in a few cases: the type of injury, the presence of vascular insufficiency or physiologic derangement, and the surgeon’s individual experience [1,15]. Due to the complexity of vascular trauma, many patients must be treated in vascular surgical centers initially not intended for pediatric care [6,7,18,19]. While the principles of arterial reconstruction and revascularization are the same in both pediatric and adult patients, there are renown essentials during open surgical repair that are worth mentioning in pediatric patients [1,7,8,14,15,17]. First, we should remember that arterial reconstruction needs more than just patency-preserving flow to adequately support vessel growth. Whether primary repair or graft interposition is performed, interrupted suture should be pursued; the smaller caliber of vessels offers less room for error, so great care should be paid to ensure meticulous technique. Interrupted sutures are much less prone to the potential “purse stringing” effect, with growth correlated with circumferential running suture. Second, when primary repair is not feasible, every effort should be made to use autologous materials for several reasons: they better accommodate the eventual mismatch in diameters, they have the potentiality to adequately adapt to the progressive growth of the repaired vessel, and they may limit the risk of superinfection that is always a potential threat in trauma settings. Third, the more pronounced vascular spasm that occurs in children and the reduced vessel size compared with adults may present technical difficulties during open surgery; the direct use of an intra-arterial vasoactive agent (e.g., topical papaverine or lidocaine) may offer some beneficial effects in such situations.

### 4.6. Endovascular Repair

In adult vascular surgery, there has been a dramatic change toward the utilization of endovascular techniques; however, endovascular technology remains limited for pediatric arterial injuries [10,17,18,19,24]. In most of the experiences noted in our review, endovascular intervention was primarily used to treat aortic injuries [10,13,21,23]; sporadically, endovascular means were used to restore vessel patency in extremities. However, as also highlighted in two of our cases, endovascular treatments may be viable and effective alternative options. Especially in unstable patients, they offer a fast temporization of bleeding, which is a primary goal in major vascular traumas [17]. If not a definitive treatment, they may act as a bridge for a subsequent definitive open surgical reconstruction. These findings also underscore that diagnostic angiography is no more worthwhile, since it should be considered only in such cases where it may be subsequently used as a therapeutic maneuver [1,3]. The most limiting aspects of endovascular repair, especially when considering stent-grafting, are access and size-specific graft limitations that restrict wide applicability of this technology on most occasions. Developing technologies such as bioresorbable stents and grafts may be promising to overcome the current growth-related challenges of endovascular techniques and prosthetic conduits. Additionally, balloon-expandable polytetrafluoroethylene and serial balloon angioplasty with drug-coated balloons-extrapolated from reports for developmental pathology such as midaortic syndrome may prove useful to facilitate conduit expansion with growth [24].

### 4.7. Mortality and Morbidity

While lower-extremity vascular injuries in the pediatric population seem to be associated with significantly lower mortality when compared to adult patients with similar injuries, our review shows a high incidence of morbidity that exceeds 10% in most of the clinical experiences [3,4,5,19]. Although a majority of the treated patients had successful revascularization, and mean limb salvage was as high as 94%, lifestyle impairment following vascular trauma may remain and mainly associated with lower extremity injuries, especially those involving the popliteal artery [3,5,9]. These figures are significantly different when considering aortic trauma, especially determined by firearm; in the large analysis of the National Trauma Databank of Barmparas et al. [1], almost 20% of children injured by a firearm did not survive and almost half of the patients who had sustained an intrathoracic vascular injury owing to a penetrating mechanism also died. In particular, when reviewing the Kids’ Inpatient Database, Tashiro et al. [13] documented that patients with traumatic shock or necessitating exploratory laparotomy had the highest associated mortality overall. However, it is interesting to note that survival of pediatric patients suffering from aortic trauma increased during the course of the years. Such recent improvements in mortality seem to be corroborated by the findings from the other single-institution case series that exhibited no mortality, and where an endovascular approach may have played a positive role to limit morbidity by reducing operative time, blood loss, and hospitalization in comparison with open repair [10,21].

### 4.8. Postoperative Management

One of the most important pending matters when treating pediatric major arterial injuries is the postoperative anti-thrombotic treatment. It goes without saying that, in general, pediatric patients with major arterial injuries that have been repaired with autologous or synthetic grafts should be considered for anti-thrombotic agents to maximize patency of the conduit. Recommendations published in the available pediatric patients’ literature have been generally extrapolated from adult experiences, being characterized by wide heterogeneity [20,21,24]. Anti-thrombotic therapy is an important consideration in the management of pediatric vascular injuries. In a study by Morão et al. [6], pediatric patients who were deemed at high risk for postoperative thrombosis were given one week of enoxaparin (1 mg/kg dosing). In a study by Kirkilas et al. [5], a multimodal regimen comprising heparin, aspirin, or enoxaparin for varying lengths of treatment was provided, thus once again highlighting the absence of clear recommendations for postoperative protocols. Aspirin was the most prescribed outpatient regimen for pediatric patients with arterial injuries [20]. However, the major drawback of the use of acetylsalicylic acid is its theoretical contraindication in patients under the age of 16 due to the correlation with a rare but serious pediatric disease called Reye’s Syndrome [25]. Anticoagulation may be considered as well, but it carries its risks and requires careful consideration in the pediatric population [5,6]. Therefore, further analyses are mandatory to determine which pharmacologic agents should be utilized, and the optimal length of treatment. 

### 4.9. Follow-Up

Another important issue is the follow-up program after major arterial injuries repair in pediatric patients. These patients have been considered at high risk for the development of complications [17]. Although the preservation of functional status after revascularization appears satisfactory from our review, with very few arterial reinterventions having been reported, follow-up compliance was meager; the established body of literature for trauma with true long-term follow-up is scant [1,3,5,8,18,26]. Therefore, the true incidence of reintervention may be underestimated, and follow-up protocol mandates robust refinement especially because pediatric patients have their whole lives ahead of them to be exposed to problems related to the index intervention.

### 4.10. Limitations

This study has several limitations. This study is essentially a narrative review and therefore does not allow direct comparison between studies, does not meet important criteria to help mitigate bias, and there was no evaluation of the selected articles for validity. Notwithstanding, considering the lack of data correlated to major arterial injuries in pediatric patients, it is a comprehensive collection of data from the largest experiences published so far.

## 5. Conclusions

Major arterial injuries in pediatric patients are rare, with an estimated overall incidence of <2% of all vascular traumas. Although the trauma-related mortality rate is lower in comparison with adult traumas, especially after extremity injuries, and limb salvage is reported to be satisfactorily higher than 94%, major morbidity has been reported, homogeneously exceeding a 10% rate. Clear recommendations regarding indication for operative repair, type of reconstruction, and postoperative management, especially for anti-thrombotic therapy, are disappointingly lacking, and expert consensus documentation is eagerly awaited.

## 6. Future Directions

- Pediatric major arterial traumas, though infrequent, are still a matter of concern in the life of vascular and trauma surgeons. This means only a few cases per surgeon, and therefore, a possible solution to improve expertise in managing vascular lesions in pediatric patients could be better training in the management of vascular trauma during the education of pediatric (vascular) surgeons.
- Future research will be directed toward studying long-term outcomes. Long-term follow-up is needed in pediatric patients treated with endovascular repair for aortic traumas. Additionally, developing technologies such as readily available conduits for the reconstruction of extremity vessels that have the ability to grow with a child could eliminate the need for multiple reoperations and long-term morbidity. Lastly, further analysis is necessary to determine which pharmacological agents should be utilized, and the optimal length of treatment.

## Figures and Tables

**Table 1:** Summary of the largest clinical experiences reporting on major arterial injuries in pediatric patients.

Authors	References	Study	Series	Cases	Incidence	Upper Extremity	Lower Extremity	SAT	Torso	Endovascular	Mortality	Morbidity	Limb Salvage	Follow-Up	Reintervention
	(n)	(Type)	(Years)	(n)	(%)	(%)	(%)	(%)	(%)	(%)	(In-Hospital)	(In-Hospital)	(%)	(Mean, Years)	(%)
Barmparas, et al.	[1]	national registry	2002–2006	1138	0.6	35.7	18.6	9.9	37.4	0	13.2	n.r.	91.8	n.r.	n.r.
Mommsen, et al.	[3]	single-center	1971–2006	44	n.r.	31.6	61.4	not included	not included	0	n.r.	18.2	81.8	n.r.	n.r.
Sciarretta, et al.	[4]	single-center	2006–2011	18	0.6	not included	100	not included	not included	0	5.5	22.2	94.5	n.r.	n.r.
Kirkilas, et al.	[5]	single-center	2008–2013	23	n.r.	73.9	26.1	not included	not included	0	0	22.0	87.0	n.r.	n.r.
Morao, et al.	[6]	single-center	2009–2015	21	0.5	75.0	25.0	not included	not included	0	0	0	100	4.3	0
Markovic, et al.	[7]	single-center	1993–2018	17	n.r.	70.6	29.4	not included	not included	0	0	11.8	94.1	2.3	6.2
Mousa, et al.	[8]	multicenter	2008–2015	149	n.r.	49.0	51.0	not included	not included	0	3	13.0	97.0	n.r.	n.r.
Rehman, et al.	[9]	single-center	2008–2018	75	4.4	56.0	44.0	not included	not included	0	n.r.	n.r.	92.6	n.r.	n.r.
Kim, et al.	[10]	single-center	2008–2019	16	n.r.	not included	not included	not included	100	6.2	0	0	n.a.	0.6	0
Tashiro, et al.	[13]	multicenter	1997–2009	468	n.r.	not included	not included	not included	100	4	35.5	n.r.	n.r.	n.r.	n.r.
Klinkner, et al.	[14]	single-center	1993–2005	103	1.1	22.3	23.3	19.4	13.5	1.0	9.7	n.r.	88.3	n.r.	n.r.
Corneille, et al.	[15]	single-center	1995–2008	116	1.4	38.7	24.1	6.9	25.9	0	12.1	12.9	97.3	n.r.	n.r.
Shah, et al.	[16]	single-center	2000–2006	42	0.4	78.4	21.6	n.r.	n.r.	0	0	7.1	100	n.r	n.r
Wang, et al.	[17]	single-center	2010–2017	23	1.6	60.9	30.4	8.7	not included	4.3	0	0	100	3.6	0
Myers, et al.	[18]	single-center	1985–1988	20	1.5	25.0	35.0	20.0	15.0	0	5.0	5.0	100	1.3	0
Kayssi, et al.	[19]	single-center	1994–2014	106	1.2	47.7	19.8	9.4	17.9	0.9	0.9	29.2	99.1	n.r.	n.r.
Shahi, et al.	[20]	multicenter	2008–2018	84	n.r.	39.3	28.6	7.1	25.0	3.6	n.r.	17.0	98.8	n.r.	17.9
Brinkman, et al.	[21]	single-center	1990–2013	17	n.r.	not included	not included	not included	100	41.1	0	n.r.	100	1.2	0

*n* = number; SAT = supra-aortic trunks; n.r. = not reported.

## Data Availability

The data underlying this article will be shared on reasonable request to the corresponding author.
